# Genetic Diversity on a Rare Terrestrial Orchid, *Habenaria linearifolia* in South Korea: Implications for Conservation Offered by Genome-Wide Single Nucleotide Polymorphisms

**DOI:** 10.3389/fpls.2022.772621

**Published:** 2022-02-24

**Authors:** Soo-Rang Lee, Tae-Young Choi, Su-Young Jung

**Affiliations:** ^1^Department of Biology Education, College of Education, Chosun University, Gwangju, South Korea; ^2^Division of Forest Biodiversity and Herbarium, Korea National Arboretum, Pocheon, South Korea

**Keywords:** *Habenaria*, genetic diversity, population structure, SNP, terrestrial orchid, endangered species

## Abstract

Monitoring intraspecific diversity offers invaluable insights on conservation practices as the variation is the product of species evolution. Accordingly, the role of population genetic diversity has drawn great attention over the last century responding to the biodiversity loss induced by a series of anthropogenic changes. Orchids are one of the most diverse, yet ironically most rapidly disappearing plant groups due to the specialized habitat preferences. Thus, population-level genetic diversity studies may offer a powerful tool for orchid conservation programs. Using the 3 restriction site-associated DNA (3RAD) approach, 2,734 genome-wide single nucleotide polymorphisms (SNPs) were isolated. With the 2,734 SNPs, we investigated genetic diversity and population structure on 72 individuals of *Habenaria linearifolia* and *Habenaria cruciformis* in South Korea. Overall, the genetic diversity was well maintained in South Korean *Habenaria*, but high F_*ST*_ values were estimated suggesting large population diversification with limited gene flow. Bayesian assignment analysis revealed a morphologically cryptic diversity pattern in Jeju Island populations, which might serve as an evolutionarily significant unit.

## Introduction

Monitoring the population-level genetic diversity for a species offers invaluable insights into conservation practices as the variation directly reflects the evolutionary and ecological process that the species has experienced (Olson et al., 2016; Mimura et al., 2017; Schierenbeck, 2017; de Kort et al., 2021). Biodiversity encompasses variations at all levels from the infraspecific to the community, yet infraspecific diversity, including population-level genetic variation, has been relatively underestimated despite its rate of loss exceeding that of higher-level diversity (Mimura et al., 2017; Kooyman et al., 2020). Within the last century, large bodies of land surfaces have been heavily exploited by human activities resulting in accelerated rates of biodiversity loss (Hautier et al., 2015; Scheffers et al., 2016). Accordingly, the role of population genetic diversity has become increasingly significant in terms of responding to biodiversity loss and subsequent conservation strategies (des Roches et al., 2021; Hohenlohe et al., 2021; Stange et al., 2021). For example, Lee et al. (2018) suggested risk in deleting a rare myrmecochorous herb, *Plagiorhegma dubium* from the Red List of South Korea based on the pattern of population genetic diversity of simple sequence repeat data despite the recent growth of census population sizes.

Orchidaceae is a cosmopolitan plant group that comprises well beyond 20,000 taxa exhibiting the most remarkable diversity of all levels from infraspecific to generic (Summerhayes and Withner, 1960; Cozzolino and Widmer, 2005). Ironically, at the same time, orchids are one of the most rapidly disappearing plant groups, which are listed heavily in the IUCN Red List, for example, more than 800 orchids are designated as threatened (Cribb et al., 2003; Wraith et al., 2020; IUCN, 2021). Coupled with intrinsic factors such as complex symbiotic relationships with specialized pollinators and mycorrhizal fungi, anthropogenic threats such as climate change, habitat destruction, and poaching expedite the rate of diversity loss in Orchidaceae (Swarts and Dixon, 2009; Phillips et al., 2020; Wraith et al., 2020). Given the current pace of climate changes, commonly required specific niche preferences of terrestrial orchids will likely expose them to high extinction vulnerability (Orchid Specialist Group [UCN/SSC], 1996; Swarts and Dixon, 2009). Local populations of terrestrial orchids are likely to become isolated and patchy under ongoing environmental changes. Consequently, the infraspecific genetic diversity would drop more quickly than the species and generic-levels diversities. Therefore, rigorous monitoring of population-level genetic diversity in terrestrial orchids is highly recommended for conservation and management plans.

*Habenaria* Willd. is one of the most diverse taxonomic groups of the terrestrial orchids distributed across the old and the new worlds, which consists of more than 800 taxa (Govaerts et al., 2010). Despite the large breadth of distribution, ∼10% of *Habenaria* species display a high vulnerability to extinction (IUCN, 2021). The proportion of threatened taxa in the genus will likely grow over time due to ongoing environmental changes and a lack of knowledge on many *Habenaria* taxa. Despite the increased vulnerability, there are only a handful of *Habenaria* taxa for which the population-level diversity pattern has been investigated (Zhang and Gao, 2017; Chung et al., 2018; Tachibana et al., 2021). *Habenaria linearifolia* (Maximowicz) Szlachetko, a rare terrestrial orchid with a narrow distribution (South Korea, E China, Japan, and Russian Far East) is restricted to bogs and wet grasslands (Lee and Choi, 2006; Chen and Cribb, 2009; Chung et al., 2018). As for many terrestrial orchids, populations of *H. linearifolia* are small, patchy, and isolated (Chung et al., 2018), which likely promotes extinction risk. Notably, the species is not on any protected species list, partly due to a lack of studies examining the infraspecific diversity status. Accordingly, an infraspecific genetic diversity study at a genomic scale might offer a diagnosis of the current level of diversity and vulnerability for *H. linearifolia*.

In this study, we surveyed genomic diversity and the spatial pattern of *H. linearifolia*, a rare terrestrial orchid in South Korea. *Habenaria cruciformis*, the most closely related species having a questionable taxonomic boundary problem with *H. linearifolia* was also included in the study. Using thousands of genome-wide single nucleotide polymorphism (SNP) markers, we aimed to (1) assess the extent of genetic diversity within and among populations; (2) evaluate gene flow among *H. linearifolia* populations and between the two species; (3) determine the presence of genetic bottlenecks; and finally (4) identify any cryptic diversity that is presently unknown. Given the specialized habitat preferences and the obligatory symbiotic relationships with pollinators and mycorrhizal fungi, we hypothesized that the populations of *H. linearifolia* have largely differentiated with low within-population genetic diversity. *Habenaria cruciformis* population might have largely diverged from all *H. linearifolia* populations with very limited gene flow if the species is an independent species with complete reproductive isolation. The results may suggest separate management units and/or assist in determining the conservation priority.

## Materials and Methods

### Study System

*Habenaria linearifolia* is 25–90 cm tall with whitish-green flowers blooming from July to September (Ohwi, 1965; Lee and Choi, 2006; Chen and Cribb, 2009). The plant is characterized by a long spur (2.5–6 cm) that is pendulous along with the shape of lateral lobes (Lee, 2007). *Habenaria linearifolia* does not clonally propagate, but it is self-compatible (Chung et al., 2018). The flowers mostly are pollinated by hawkmoths and butterflies (Chung et al., 2018). Mature capsules (1.5–2 cm long) contain numerous small seeds. The species is a diploid with the chromosome number, 2*n* = 28 (Chen and Cribb, 2009). *Habenaria cruciformis*, a terrestrial orchid endemic to South Korea, is 50–90 cm tall and distinguished from *H. linearifolia* by shorter spur length and recurved lateral lobes (Lee, 2007). The two species share high morphological affinities and ecological characteristics.

### Sample Collection and DNA Isolation

We collected samples of *H. linearifolia* and *H. cruciformis* from nearly all known local populations. In total, 72 *H. linearifolia* samples from 10 populations and 10 samples of *H. cruciformis* from one location were collected (Table 1). Given the species’ rarity in South Korea, we did not provide detailed GPS coordinates. Fresh leaf tissues were collected from the field and preserved in a plastic zip-lock bag with silica-gel desiccant until the DNA extractions. We only sampled one adult plant from each population for voucher specimen preparation to minimize the potential effect of sampling. Genomic DNA extraction was performed with the dried leaf tissues using DNeasy Plant Mini Kit (Qiagen, Hilden, Germany) following the manufacturer’s protocol. The quality of the isolated DNA was inspected by visualizing the DNA on a 1% agarose gel through gel electrophoresis. For the DNA quantification, a Qubit 4 Fluorometer (Thermo Fisher Scientific, Waltham, MA, United States) was employed. After the quantity check, the extracted DNAs were stored at −20°C.

**TABLE 1:** Location information of *Habenaria linearifolia* and *Habenaria cruciformis* sampling sites.

Species	Location	Abbreviation	Cluster	*N*	Lon	Lat	He[± sd]	Ho[± sd]	Na[± sd]	Na_Rare	Polymorphic loci (%)
*H. linearifolia*	Aphaedo, Jeonnam, South Korea	AH	Deme 1	4	126***	34***	0.26 [0.004]	0.25 [0.005]	1.670 [0.010]	1.58	68.6
	Hwasoon, Jeonnam, South Korea	HS	Deme 1	2	127***	35***	0.19 [0.004]	0.24 [0.006]	1.425 [0.010]	1.51	44.7
	Mooan, Jeonnam, South Korea	JN	Deme 1	13	126***	35***	0.28 [0.004]	0.27 [0.004]	1.787 [0.008]	1.55	81.0
	Gijang, Gyeongnam, South Korea	IG	Deme 2	8	129***	35***	0.29 [0.004]	0.30 [0.004]	1.810 [0.008]	1.5	74.8
	Jangsan, Gyeongnam, South Korea	JS	Deme 2	7	129***	35***	0.26 [0.004]	0.26 [0.005]	1.748 [0.008]	1.47	67.8
	Dongdaesan, Gyeongnam, South Korea	DD	Deme 2	7	129***	35***	0.24 [0.004]	0.27 [0.005]	1.678 [0.009]	1.51	68.4
	Namwon, Jeju, South Korea	NW	Deme 3	5	126***	33***	0.26 [0.004]	0.30 [0.005]	1.684 [0.009]	1.58	84.2
	Halla, Jeju, South Korea	JJ	Deme 3	9	126.***	33.***	0.30 [0.003]	0.25 [0.004]	1.842 [0.007]	1.44	67.3
	Sanghyo, Jeju, South Korea	SH	Deme 4	7	126***	33***	0.23 [0.004]	0.23 [0.004]	1.673 [0.009]	1.52	78.7
	Inje, Gangwon, South Korea	GW	Deme 5	5	128.***	38.***	0.22 [0.004]	0.21 [0.004]	1.587 [0.009]	1.44	58.7
*H. cruciformis*	Youngwol, Gangwon, South Korea	YW	Deme 6	10	128***	37***	0.29 [0.003]	0.29 [0.004]	1.851 [0.007]	1.55	85.1

*N, Sample size. Lat and Lon refer to geographic coordinates. Na- mean number of alleles, Na_Rare- mean number of alleles adjusted by population sizes, He- mean expected heterozygosity, Ho- mean observed heterozygosity, sd-standard deviation.*

***Because H. linearifolia is endangered, some of the coordinates were censored.

### Restriction Site-Associated DNA sequencing Library Preparation and Sequencing

For genotyping, we employed 3RAD (Bayona-Vásquez et al., 2019), which is a reduced representation approach modified from the well-known ddRADseq (Peterson et al., 2012). The method uses a third restriction enzyme to cut adapter dimers to increase the adapter ligation efficiency, likely improving the yield of the amplified reads. For the 3RAD library preparation, we first digested the genomic DNA using *Eco*RI-HF and *Xba*I. *Nhe*I was added for dimer cleaving (all enzymes from Thermo Fisher Scientific, Waltham, MA, United States). Index adapters (BadDNA, University of Georgia, Athens, GA, United States) were ligated in 15 μl reactions containing 100 ng DNA, 8 μl master mix, and 1 μl of each 5 μM adapter. We prepared the master mix to make sure each 15 μl reaction volume contained 1 reaction of each restriction enzyme and 1.5 μl 10× FastDigest Buffer. The digestion and ligation were administered simultaneously. We then incubated the samples in a thermal cycler for 15 min at 37°C. The ligation mixture was prepared as follows: 0.5 μl 10× Ligase Buffer, 100 units T4 DNA ligase, 1.5 μl 10 mM ATP, and 2 μl ultra-pure water. We added 5 μl ligase mixture to each sample and subsequently incubated at 22°C for 20 min and 37°C for 10 min. The process was repeated two times, then incubated at 80°C for another 20 min. We ran a test PCR to check the ligation results using Bioneer multiplex premix. A 20 μl reaction was made containing 1 μl each of iTru5 and iTru7 primers and 1 μl of adaptor-ligated DNA fragments. Samples were amplified using the following thermal cycler profile: 95°C for 10 min; 35 cycles of 95°C for 30 s, 60°C for 1 min, 72°C for 30 s; and 72°C for 5 min. We then pooled 5 μl of the adaptor-ligated fragments from each sample in a 1.5 ml tube. Pooled samples were purified using a 1:1.8 mixture of AmPure XP magnetic beads (Beckman Coulter, Brea, CA, United States). We washed the samples with 70% EtOH, which then sat at room temperature for 15 min to air dry and resuspended with 60 μl TE buffer. Subsequently, a 50 μl reaction was prepared containing 10 μl of the pooled DNA fragments, 5 μl of 5 μM iTru5 primer, 5 μl of 5 μM iTru7 primer, 10 μl 5× HF Buffer, 1.5 μl of 10 μM dNTP, and 1 unit of Phusion DNA polymerase. We then amplified the samples: 98°C for 2 min; 7 cycles of 98°C for 20 s, 60°C for 15 s, 72°C for 30 s; and 72°C for 5 min. Another sample clean-up was administered by using a 1:1.8 mixture of AmPure beads, washed twice with 70% EtOH and resuspended in 60 μl TE. For the targeted size selection of 500-bp fragments (± 10%), we used Pippin Prep (Sage Science, Beverly, MA, United States). Final amplification was conducted using 50 μl PCRs containing 5 μl of size-selected DNA, 3 μl of 5 μM P5 primer, 3 μl of 5 μM P7 primer, 1.5 μl of 10 μM dNTP, 10 μl 5× HF Buffer, and 1 unit of Phusion DNA Polymerase. The thermal cycler profile was as follows: 98°C for 2 min, 98°C for 20 s, 61°C for 15 s, and 72°C for 45 s for 12 cycles; and 72°C for 5 min. We did a final clean-up for the amplicons with a 1:1.8 mixture of AmPure beads, washed twice with 70% EtOH and resuspended in 35 μl ultra-pure water. We evaluated the completed 3RAD library for quality and quantity in Agilent 2100 Bioanalyzer (Agilent Technologies, Santa Clara, CA, United States). We ran the library on an Illumina HiSeq X-10 using 2 × 150 paired-end sequencing at Macrogen Inc., South Korea.

### SNP Calling

All sequence data produced were demultiplexed, trimmed, and processed in Stacks version 2.41 (Rochette et al., 2019). For the quality check of the raw reads, a Phred score of 10 was applied using the process_radtag function. We assembled the Restriction site-associated DNA (RAD) loci *de novo* due to the lack of a fully assembled and annotated reference genome. To assemble catalogs from the quality-filtered reads, we set the parameters as −m 3 and −M 3 in the ustacks function, then we allowed a maximum of one mismatch between sample loci (−*n* 1, cstacks function) following Paris et al. (2017). After the catalog construction, we called SNPs by Population software implemented in STACKS. SNP loci were identified if the loci were present in at least 80% of the samples within each population, and shared by at least 5 populations (−*p* 5 and −*r* 0.80). We further screened the SNP loci that significantly departed from the Hardy–Weinberg Equilibrium (threshold = *P* < 10e^–6^; Li, 2011; Hess et al., 2013) to avoid the loci with extreme heterozygosity and assembly errors in PLINK 1.9 (Purcell et al., 2007). To ensure the independence of alleles, SNP loci in strong linkage disequilibrium (*r*2 > 0.8; Tian et al., 2009) were purged using PLINK. We also removed the genotypes with more than 30% missing calls and SNP loci with a minor allele frequency of ≤0.05 were pruned from the data set in TASSEL 5.0 (Glaubitz et al., 2014).

### Data Analysis

After the SNP call steps, five *H. linearifolia* genotypes that failed the quality checks were eliminated from the downstream analyses. The number of SNP loci used in the downstream analyses differs among the types of analysis due to the dependent assumption and the computational complexity. Using 2,734 SNPs, three within-population genetic diversity parameters-number of alleles (Na), expected heterozygosity (He), observed heterozygosity (Ho), and percentage of polymorphic loci were computed in GenAlex 6.5 (Peakall and Smouse, 2012). As the population sizes vary, Na was adjusted using rarefaction curves to account for unequal sizes of 11 local populations (Table 1; Kalinowski, 2004) in HP-Rare (Kalinowski, 2005). Population divergence was estimated as pairwise F_*ST*_ between all 11 population pairs based on 2,734 SNPs in Arlequin version 3.5 with 1,000 permutations for the significance test (Excoffier and Lischer, 2010). Through analysis of molecular variance (AMOVA), the total genetic variance was hierarchically partitioned by regions (as shown in the cluster in Table 1 for the region info), populations, and individuals in Arlequin. We resampled 1,000 times from the data through permutation for the statistical significance. Isolation by distance (IBD) was assessed using a Mantel test based on the correlation between linearized pairwise genetic divergences (F_*ST*_) and Euclidean distances (Rousset, 1997) in GenAlex. We employed 1,000 random permutations to test statistical significance for the correlation.

The cluster assignment pattern was examined by two approaches, principal coordinate analysis (PCoA) by GenAlex and Bayesian model-based clustering by STRUCTURE v. 2.3.4 (Pritchard et al., 2000). For the two clustering analyses, 2,734 SNPs were used. We performed the PCoA on Nei’s (1972) genetic distance estimated from all 72 individuals. For the STRUCTURE analysis, we employed the admixture for ancestry model and the correlated allele frequency model (Falush et al., 2003). We repeated ten independent runs with 1,000,000 MCMC iterations following 100,000 burn-in steps for the Ks from 1 to 10. The number of clusters (K) explaining the data the most was explored by ΔK computation (Evanno et al., 2005) in STRUCTURE HARVESTER Web v. 0.6.94 (Earl and VonHoldt, 2012). We then summarized the ancestry coefficients of the 77 genotypes estimated from 10 STRUCTURE replicates using CLUMPP v. 1.1.2 (Jakobsson and Rosenberg, 2007) with the greedy option. DISTRUCT v. 1.1 (Rosenberg, 2004) was used for the graphical representation of the ancestry result.

One of the study aims was to examine the level of gene flow among populations with geographic barriers and between the species with a taxonomic boundary issue. To address the goal, migration rates among populations and between the two species were measured as a proxy for the gene flow. Given the size of the genomic datum, computational challenges and parameter calculation biases were highly expected. Accordingly, we redefined the groups based on the genetic clusters inferred from the STRUCTURE (Figure 3; see Table 1 for the redefined cluster acronyms). We assessed the migration rates among the six rearranged clusters including five regional clusters (hereafter called demes) of *H. linearifolia* and one cluster of *H. cruciformis*. For the analysis, we randomly selected 303 unlinked SNPs to ease the computational complications. We utilized a coalescent-based approach implemented in MIGRATE- N 3.6.11 (Beerli and Palczewski, 2010) for the migration rate calculation. We calculated M (= m/μ, m–an immigration rate per generation and μ–a mutation rate) assuming asymmetric migrations among the clusters and θ (4Ne μ, where Ne = effective population size; μ = mutation rate; Beerli and Palczewski, 2010). Before the actual run, we did a test run with a small subset (48SNPs) of the data to determine the range of parameters. Given the geographic separation of Jeju Island from the peninsula and the test run result, we assumed one-way migration for the Jeju populations (the main land Jeju Island). A Bayesian approach was employed and two independent runs were repeated with a constant mutation rate across all loci. We set the prior distribution for the parameters θ and M as uniform with minimum, mean, maximum, delta, and bin values of 0, 0.05, 0.1, 0.01, and 1,500 and 0, 500, 1,000, 100, and 1,500, respectively. Each repeated run was as follows: 1 long chain with four heating chains (1, 1.5, 3, and 106). We applied 2,500,000 iterations with an increment size of 50 after a burn-in of 10,000.

**FIGURE 1:**
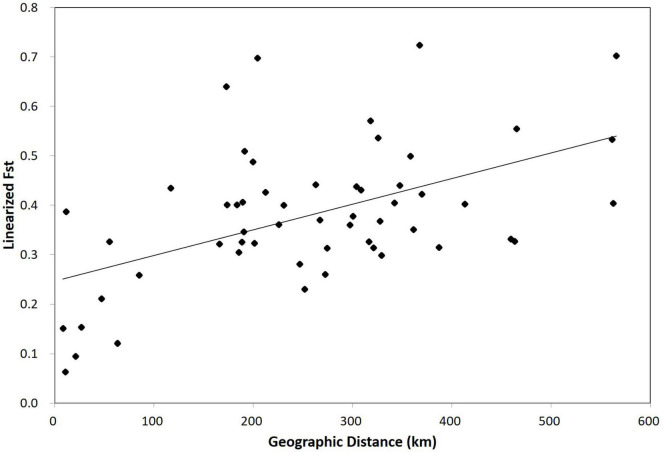
A plot of the correlation between geographic distance (Euclidean) and F_*ST*_ of 55 *Habenaria linearifolia* and *Habenaria cruciformis* population pairs in South Korea. A significant isolation by distance pattern was identified (*r* = 0.52, *P* < 0.01).

**FIGURE 2:**
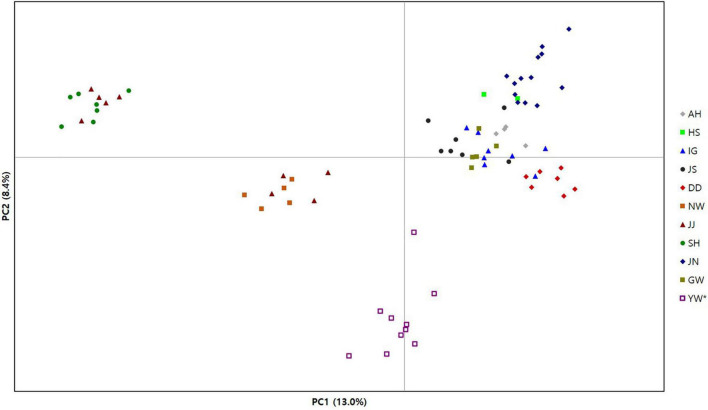
Principal components analysis plot for 72 individual genotypes of *Habenaria linearifolia* and *Habenaria cruciformis*. The first two variance components from 2,734 SNPs were plotted. Table 1 shows abbreviations of population locations and sample sizes.

**FIGURE 3:**
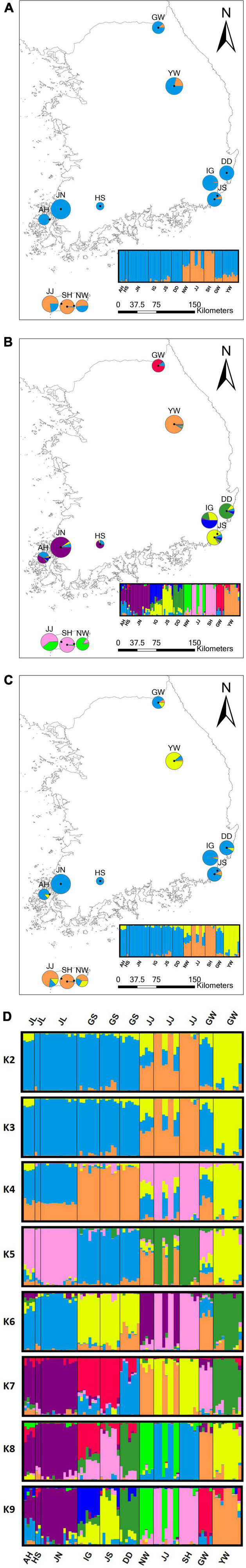
Bayesian model-based assignment analysis of 2,734 SNPs for 10 populations of *Habenaria linearifolia* and one population of *Habenaria cruciformis*. Pie charts on the map show the frequency of each cluster in a population. See Table 1 for abbreviations of population locations and sample sizes. **(A)** K2, the best K plot; **(B)** K3 plot; **(C)** K9 plot; and **(D)** K2–K9 bar plot. Populations are separated by vertical black lines.

We examined whether the populations have experienced recent bottlenecks with 303 unlinked SNPs to ease the computational challenges. Populations at approximate mutation-drift equilibrium show an equal probability of a heterozygosity excess or a heterozygosity deficit. We evaluated an excess of heterozygosity followed by Cornuet and Luikart (1996) in the software BOTTLENECK (Piry et al., 1999). BOTTLENECK was run with the infinite allele model (IAM) and the step-wise mutation model (SMM). For statistical robustness, we applied the sign test implemented in BOTTLENECK. The presence of a mode shift from the equilibrium state in allele frequencies was also determined.

## Results

In total, the library produced ∼108 Gbp with an average GC content of 39%. From the initial SNP call, we isolated over 91K SNPs. After a series of pruning steps with the thresholds described, 2,734 SNPs were retained for the downstream analyses. Overall genetic diversity of *H. linearifolia* varied across populations although the differences were not significant (Table 1). The number of alleles adjusted by the population size ranged from 1.44 (GW and JJ; see Table 1 for the population acronyms) to 1.58 (AH and NW). The mean He (= 0.26) and percentage of polymorphic loci (mean = 70.9) were the lowest in HS at which the collected sample was the smallest (Table 1). Among-population level genetic divergence measured by F_*ST*_ varied largely across the population pairs ranging from 0.07 (JJ/SH) to 0.30 (HS/SH; Table 2). All F_*ST*_ values were statistically significant at the *P* < 0.01 level.

**TABLE 2:** Estimated pairwise F_*ST*_ values from 2,734 SNPs among 10 *Habenaria linearifolia* populations and one *Habenaria cruciformis* population in South Korea.

	AH	HS	IG	JS	DD	NW	JJ	SH	JN	GW	YW
AH	0.000										
HS	0.207	0.000									
IG	0.152	0.211	0.000								
JS	0.180	0.219	0.096	0.000							
DD	0.189	0.250	0.125	0.160	0.000						
NW	0.204	0.265	0.168	0.192	0.224	0.000					
JJ	0.182	0.226	0.155	0.156	0.199	0.092	0.000				
SH	0.253	0.297	0.220	0.217	0.270	0.186	0.069	0.000			
JN	0.111	0.146	0.123	0.142	0.161	0.179	0.153	0.220	0.000		
GW	0.214	0.241	0.199	0.203	0.214	0.244	0.204	0.273	0.174	0.000	
YW*	0.189	0.250	0.170	0.178	0.188	0.170	0.160	0.226	0.175	0.208	0.000

Table 1 shows abbreviations of population locations and sample sizes. All values were statistically significant at the P < 0.05 level. *Habenaria cruciformis population.

According to AMOVA, ∼60% of genetic variance was attributable to variations within individuals (Table 3). A fairly large proportion of total genetic variance (∼18%) was partitioned among regional groups redefined by the Bayesian assignment analysis (Table 3). Genetic variance among populations within each region was smaller than that derived from among regions (Table 3). We found a significant IBD pattern from the Mantel test result (*r* = 0.52; *P* < 0.01; Figure 1).

**TABLE 3:** Analysis of molecular variance (AMOVA) results of 2,734 SNPs among 10 *Habenaria linearifolia* populations and one *Habenaria cruciformis* population in South Korea.

Source	Sum of squares	Variance components	Percentage of variation	Fixation index
Among groups (FCT)	16438.137	92.454	17.157	0.172
Among population within groups (FSC)	7478.536	68.195	12.655	0.153
Among individuals within populations (FIS)	26358.319	31.187	5.787	0.082
Within individuals (FIT)	26163.500	347.028	64.400	0.356

*All variance components were statistically significant (P < 0.01).*

Our clustering results of both PCoA and STRUCTURE exhibited similar patterns, which largely separates three populations in Jeju Island from the remaining eight populations (Figures 2, 3). The PCoA plot of PC1 and PC2 divided the populations into four clusters primarily by the PC1 axis which explains about 13% of the total variance (Figure 2). Notably, PC1 separates the three Jeju populations of *H. linearifolia* into two clusters, whereas one *H. cruciformis* population represented as YW was isolated from the rest of the populations exclusively by the PC2 axis (∼8%; Figure 2). The best K determined by ΔK was *K* = 2 (Supplementary Figure 1); however, we plotted *K* = 2–9 and presented the bar charts here to explore the admixture patterns as the number of K clusters increased (Figure 3). At *K* = 2, three Jeju populations formed a regional deme sharing genotype frequencies represented mostly by an orange ancestry with a blue ancestry admixed (Figure 3A). As the number of the ancestry groups grew to three (*K* = 3), *H. cruciformis* started exhibiting a unique ancestry pattern with a minor portion of admixture (Figure 3B). When the K increased to nine, the pattern of the shared ancestry reflected the geography such that *H. linearifolia* populations formed four regional demes, that is, Jeonnam, Gyeongnam, Jeju, and Gangwon (Figures 3C,D).

The indirect measures of gene flow estimated from Migrate-N greatly varied among the demes and were somewhat consistent with the clustering patterns (Table 4). Overall, the gene flow among the regional demes showed asymmetry, in particular, it was the strongest between demes 1 (JN) and 5 (GW) (5 → 1 M = 0.258; 1 → 5 M = 0.014; Table 4). Deme 5 (GW; see Table 1 for the deme information) appeared to migrate much more to the other demes while it received a very limited number of immigrants from the rest of the demes (Table 4). However, the statistical support of the rates of emigration to the other demes from Deme 5 was weak (Table 4). Deme 1, Deme 2, and Deme 3 showed a rather high migration rate into the remaining demes (∼0.2–0.3/generation/site; Table 4). Based on the Bottleneck results, all populations have experienced significant reductions in population sizes with-in the recent past (Table 5).

**TABLE 4:** Results for tests of recent bottlenecks *Habenaria linearifolia* and *Habenaria cruciformis* populations.

Population	*P* (Sign test)		Mode shift
	IAM	SMM	
AH	0.000	0.005	YES
JN	0.000	0.000	YES
IG	0.000	0.000	YES
JS	0.000	0.000	YES
DD	0.000	0.000	YES
NW	0.000	0.000	YES
JJ	0.000	0.000	YES
SH	0.000	0.000	YES
GW	0.000	0.000	YES
YW	0.000	0.000	YES

*P-values from sign test for excess or deficit of heterozygosity across 303 unlinked SNPs loci under IAM and SMM mutation model.*

**TABLE 5:** The migration rates computed from the thetas (mutation scaled effective population sizes) for five demes of *Habenaria linearifolia* and *Habenaria cruciformis* (deme 6).

Group 1	Group 2	Migration
Gangwon, Deme 5	Jeonnam, Deme 1	0.258 (0.125, 0.349)
Gangwon, Deme 5	Gyeongnam, Deme 2	0.291 (0.150, 0.382)
Gangwon, Deme 5	Jeju, Deme 3	0.222 (0.093, 0.350)
Gangwon, Deme 5	Sanghyo, Deme 4	0.014 (0.000, 0.062)
Gangwon, Deme 5	*H. cruciformis*, Deme 6	0.142 (0.000, 0.253)
Gyeongnam, Deme 2	Jeonnam, Deme 1	0.260 (0.125, 0.348)
Gyeongnam, Deme 2	Jeju, Deme 3	0.222 (0.093, 0.348)
Gyeongnam, Deme 2	Sanghyo, Deme 4	0.019 (0.000, 0.062)
Gyeongnam, Deme 2	Gangwon, Deme 5	0.014 (0.000, 0.033)
Gyeongnam, Deme 2	*H. cruciformis*, Deme 6	0.142 (0.000, 0.253)
Jeju, Deme 3	Sanghyo, Deme 4	0.074 (0.000, 0.062)
Jeonnam, Deme 1	Gyeongnam, Deme 2	0.293 (0.153, 0.382)
Jeonnam, Deme 1	Jeju, Deme 3	0.223 (0.093, 0.348)
Jeonnam, Deme 1	Sanghyo, Deme 4	0.019 (0.000, 0.062)
Jeonnam, Deme 1	Gangwon, Deme 5	0.014 (0.000, 0.033)
Jeonnam, Deme 1	*H. cruciformis*, Deme 6	0.142 (0.000, 0.253)
Sanghyo, Deme 4	Jeju, Deme 3	0.083 (0.018, 0.183)

*The values in parentheses are the 95% CI.*

## Discussion

Terrestrial orchids, one of the species-rich groups, are rapidly disappearing from the earth due to their specialized habitat preference and their complex symbiotic relationships with mycorrhizal fungi and pollinators (Swarts and Dixon, 2009; Phillips et al., 2020; Wraith et al., 2020). Under the rapid and ongoing biodiversity loss induced by anthropogenic environmental changes, the reduction in orchid diversity is likely accelerated. By monitoring the population-level genetic diversity of two rare terrestrial orchids, we revealed three major findings. First, the orchid populations have experienced genetic bottlenecks and were genetically isolated with a rather low gene flow, which might suggest the possibility of local extinction (Tables 2, 4). Despite the limited gene flow and reductions in population sizes, the within-population genetic diversity in *H. linearifolia* is comparable to the other terrestrial orchids (Table 1). Finally, we found cryptic variation in the regional populations on Jeju Island from the Bayesian assignment analysis that was not shared with the inland populations (Figure 3).

Overall, the diversity of 2,734 SNPs observed in *H. linearifolia* and *H. cruciformis* appeared to be comparable or even greater to that estimated for other terrestrial orchids [Table 1; *Pleione formosana*, Ho = 0.27 (Chao et al., 2021); and *Epipactis helleborine* Na = 1.21, He = 0.054 (Jacquemyn et al., 2020)]. The amount of genetic variability harbored in a rare orchid such as *H. linearifolia* was surprising considering the population bottlenecks we found (Table 5). In addition, it is noticeable that the high molecular variation was observed only in the degree of heterozygosity and not in allelic richness (Table 1). Populations that have experienced a sudden demographic crash are expected to be low in genetic variation due to the massive loss of alleles resulting from genetic drift, yet the decline of genetic diversity may not be prominent if the bottleneck was a single event (Nei et al., 1975; Carson, 1990). Given the lengthy longevity of orchids, the one migrant per generation rule (Mills and Allendorf, 1996) might alternatively be the causal mechanism for the maintenance of diversity. However, migration rates lower than one (mean migration rate = 0.14; Table 4) undermines the probability of the hypothesis. The high heterozygosity observed might also be resulted from hybridization among multiple lineages. Indeed, Thompson et al. (2010) stressed the importance of hybridization as a source of genetic variation for rare plant species. However, our data were not equipped to test the possibility as we could not sample all regional lineages due to the inaccessibility of some northern and island populations. Further study may expand the sample sizes and determine the true mechanism for the maintenance of genetic variability.

Pairwise genetic divergences values (mean pairwise F_*ST*_ ≈ 0.2) were greater than the ones observed in many rare orchids [*Spiranthes spiralis*, mean F_*ST*_ = 0.02 (Machon et al., 2003); *Caladenia huegelii*, F_*ST*_ = 0.047 (Swarts et al., 2009); *Orchis purpurea*, F_*ST*_ = 0.09 (Jacquemyn et al., 2020)], which clearly indicate large population divergence of *H. linearifolia* (Table 2). Although reduced gene flow has been speculated based on the rarity and narrow distribution of most orchids, a recent meta-analysis revealed rather contrasting patterns of gene flow (Phillips et al., 2012). Considering the capability of long-distance dispersal resulting from the minute size and massive quantity of seeds in orchids, moderate-to-high gene flow found in the meta-analysis indeed is not surprising. *Habenaria* orchids also produce thousands of dust-like seeds (Chen and Cribb, 2009), thus the large population differentiation observed here for *H. linearifolia* was unexpected. The increased population divergence might be attributed to the limited gene flow we observed in South Korean *Habenaria* (Table 4). The minute orchid seeds may travel far, but the actual movement distance will vary for each seed produced and is highly dependent on the neighboring vegetation height (Jersáková and Malinová, 2007; Brzosko et al., 2017). As South Korean *Habenaria* mostly resides in grasslands surrounded by tall grasses, low gene flow limited by seed dispersal seems likely. Alternatively, such population diversification could also arise by a combination of strong genetic drift and natural selection (Gentry and Dodson, 1987; Tremblay et al., 2005). The effect of genetic drift is expected to some extent, however, with the current data set, it is uncertain how natural selection could impact the process of population diversification. To tease apart the role of limited gene flow from the contribution of drift and selection on population diversification, empirical studies assessing selection such as common garden experiments are needed.

Notably, Bayesian clustering analysis revealed an unexpected assignment pattern. Based on the best K (*K* = 2), 2,734 SNPs have the eleven populations of *H. linearifolia* and *H. cruciformis* assigned to two demes consisting of the inland deme and the Jeju Island deme (Figure 3A). It is rather surprising that *H. cruciformis* represented by the Youngwol population was not separated from the inland populations of *H. linearifolia*. As the cluster number, K, grows by 3, *H. cruciformis* started being divided from *H. linearifolia* showing a distinctive pattern of genotype frequency. However, it is noticeable that Jeju populations also exhibited a rather unique pattern of genotype frequency with some level of admixture in K3 (Figure 3B). *Habenaria cruciformis* was first reported by Ohwi (1951) based on the morphological aforementioned characters (the spur length and the shape of lateral lobes). On the other hand, Jeju populations were never recognized as independent taxon due to a lack of measurable morphological differences. Except for the Jeju populations, most *H. linearifolia* populations diversified following a significant IBD pattern (Figure 1).

Due to contemporary and past evolutionary processes such as long-term population isolation, single species might diverge into multiple evolutionarily significant units [ESUs; refer (Fraser and Bernatchez, 2001) for the terminology details] or even harbor a cryptic species (Bickford et al., 2007; Warner et al., 2015; Hill et al., 2016; Martin et al., 2016). With the recent advancements of modern molecular techniques, signatures of ESUs and cryptic species have been mounting. Despite the high morphological similarities, our molecular data exhibited clear separation of the island deme from both inland *H. linearifolia* and *H. cruciformis* suggesting a possibility of cryptic ESU or even a species. Delimitation of the formerly undescribed ESUs and/or cryptic species provides critical insights by identifying genetically cohesive units. To confirm the emergence of a cryptic taxon, a rigorous examination of morphological characters and a phylogenetic verification should be added. Although cautions should be taken, our data highly suggest that the Jeju Island deme must be considered as an important ESU.

The genetic diversity pattern we observed indicated that the species has been experiencing some level of diversity loss and isolation among populations with low gene flow. *Habenaria linearifolia* requires a specific type of habitat (wet meadows and bogs) that is currently shrinking with the anthropogenic interferences (Ohwi, 1965; Essl et al., 2012). Without appropriate conservation strategies, the species distribution and size of each population are likely going to diminish even further. For the conservation practices, we suggest building both close monitoring and protection programs, particularly for the newly discovered ESUs. Preparation for *ex situ* conservation, such as collecting and preserving seeds, is also recommended along with intense ecological monitoring.

## Data Availability Statement

The original contributions presented in the study are publicly available. This data can be found here: National Center for Biotechnology Information (NCBI) BioProject database under accession number PRJNA758713.

## Author Contributions

S-RL and S-YJ conceived the research idea and designed the experiments. S-YJ got the funding and revised the manuscript. T-YC conducted library preparation and related laboratory work. S-RL and T-YC performed genetic and statistical analyses. S-RL wrote the manuscript. All authors contributed to the article and approved the submitted version.

## Conflict of Interest

The authors declare that the research was conducted in the absence of any commercial or financial relationships that could be construed as a potential conflict of interest.

## Publisher’s Note

All claims expressed in this article are solely those of the authors and do not necessarily represent those of their affiliated organizations, or those of the publisher, the editors and the reviewers. Any product that may be evaluated in this article, or claim that may be made by its manufacturer, is not guaranteed or endorsed by the publisher.
