# Supplementary material for: Genetic Diversity on a Rare Terrestrial Orchid, Habenaria linearifolia in South Korea: Implications for Conservation Offered by Genome-Wide Single Nucleotide Polymorphisms
Source: Front Plant Sci. 2022 Feb 24;13:772621. doi: 10.3389/fpls.2022.772621 (PMC8907889; doi:10.3389/fpls.2022.772621)
Supplement: Supplementary file 1 [file Image_1.pdf]

Supplementary Material

Genetic diversity on a rare terrestrial orchid, *Habenaria linearifolia* in Korea: implications for conservation offered by genome-wide SNPs

Soo-Rang Lee[1*], Tae-Young Choi[1], Su-Young Jung[2]

---

[1]Department of Biology Education, College of Education, Chosun University, Gwangju 61452, Republic of Korea

[2] Division of Forest Biodiversity and Herbarium, Korea National Arboretum, Pocheon 11186, Republic of Korea

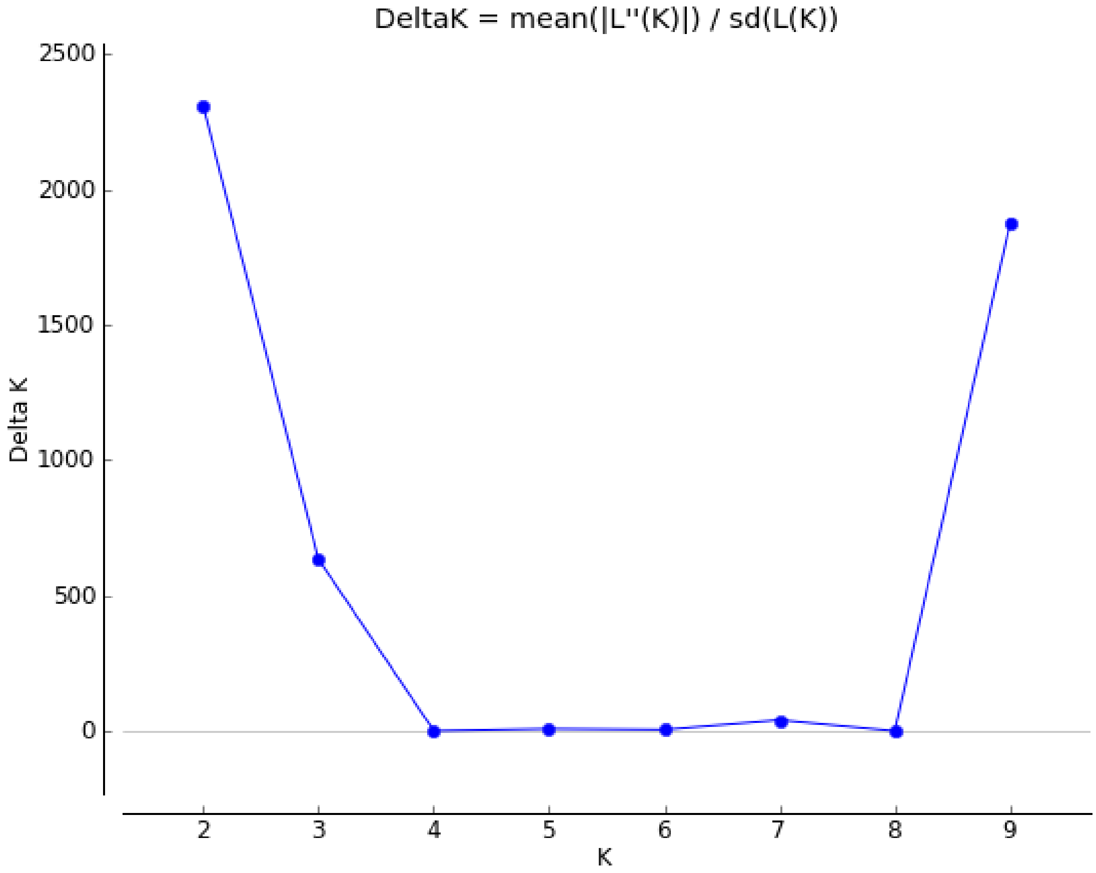

Supplementary Figure 1. Summary of delta Ks. The K values of each K cluster from 2 to 9 were computed by the method of Evanno et al. (2005).
